# α-Lipoic Acid Reduces Iron-induced Toxicity and Oxidative Stress in a Model of Iron Overload

**DOI:** 10.3390/ijms20030609

**Published:** 2019-01-31

**Authors:** Giuseppina Camiolo, Daniele Tibullo, Cesarina Giallongo, Alessandra Romano, Nunziatina L. Parrinello, Giuseppe Musumeci, Michelino Di Rosa, Nunzio Vicario, Maria V. Brundo, Francesco Amenta, Margherita Ferrante, Chiara Copat, Roberto Avola, Giovanni Li Volti, Antonio Salvaggio, Francesco Di Raimondo, Giuseppe A. Palumbo

**Affiliations:** 1 Department of Biomedical and Biotechnological Sciences, University of Catania, 95123 Catania, Italy; giusicamiolo.89@gmail.com (G.C.); d.tibullo@unict.it (D.T.); g.musumeci@unict.it (G.M.); chitotriosidase@gmail.com (M.D.R.); vicarionunzio@gmail.com (N.V.); ravola@unict.it (R.A.); 2 EuroMediterranean Institute of Science and Technology, 90139 Palermo, Italy; 3 Department of Medical and Surgical Specialties, Hematology Section, University of Catania, 95125 Catania, Italy; cesarinagiallongo@yahoo.it (C.G.); sandrina.romano@gmail.com (A.R.); lauraparrinello@tiscali.it (N.L.P.); diraimon@unict.it (F.D.R.); 4 Department of Biological, Geological and Environmental Science, University of Catania, 95129 Catania, Italy; mvbrundo@unict.it; 5 Section of Human Anatomy, School of Medicinal and Health Products Sciences, University of Camerino, Via Madonna delle Carceri 9, 62032 Camerino, Italy; francesco.amenta@unicam.it; 6 Department of Medical, Surgical Sciences and Advanced Technologies “GF Ingrassia”, University of Catania, 95123 Catania, Italy; marfer@unict.it (M.F.); chiaracopat@hotmail.com (C.C.); palumbo.ga@gmail.com (G.A.P.); 7 Experimental Zooprophylactic Institute of Sicily, 95125 Catania, Italy; antonio.salvaggio@izssicilia.it

**Keywords:** iron, alpha lipoic acid, oxidative stress, toxicity, zebrafish

## Abstract

Iron toxicity is associated with organ injury and has been reported in various clinical conditions, such as hemochromatosis, thalassemia major, and myelodysplastic syndromes. Therefore, iron chelation therapy represents a pivotal therapy for these patients during their lifetime. The aim of the present study was to assess the iron chelating properties of α-lipoic acid (ALA) and how such an effect impacts on iron overload mediated toxicity. Human mesenchymal stem cells (HS-5) and animals (zebrafish, *n* = 10 for each group) were treated for 24 h with ferric ammonium citrate (FAC, 120 µg/mL) in the presence or absence of ALA (20 µg/mL). Oxidative stress was evaluated by reduced glutathione content, reactive oxygen species formation, mitochondrial dysfunction, and gene expression of heme oxygenase-1b and mitochondrial superoxide dismutase; organ injury, iron accumulation, and autophagy were measured by microscopical, cytofluorimetric analyses, and inductively coupled plasma‒optical mission Spectrometer (ICP-OES). Our results showed that FAC results in a significant increase of tissue iron accumulation, oxidative stress, and autophagy and such detrimental effects were reversed by ALA treatment. In conclusion, ALA possesses excellent iron chelating properties that may be exploited in a clinical setting for organ preservation, as well as exhibiting a good safety profile and low cost for the national health system.

## 1. Introduction

Iron plays a pivotal role in various metabolic pathways encompassing a full range of cellular processes, such as energy metabolism and DNA synthesis, and serves as a cofactor for many enzymes, either nonheme iron-containing proteins or hemoproteins. In humans, total iron content is maintained within a range of 200–1500 mg by regulating intestinal iron absorption and metabolic recycling since no excretory mechanisms exist [1,2]. On the other hand, iron may also exhibit toxic properties when present in its free form [3]. In fact, because of its ability to participate in redox reactions, iron is a powerful catalyst for the formation of hydroxyl radicals from reduced forms of O_2_ [4]. Therefore, in order to avoid such toxic effects, iron is usually complexed with proteins as it occurs in serum, where it is mainly associated with transferrin and stored in the intracellular compartment where it is vehiculated by chaperones or stored by ferritin [5].

Different clinical conditions are associated with iron overload, and they are classified as primary or secondary forms depending on whether the overload results from a primary defect in the regulation of iron balance or it is secondary to other genetic or acquired disorders [6]. Inherited primary iron overload syndromes include hemochromatosis, caused by mutations of different genes, such as homeostatic iron regulator, hemojuvelin, hepcidin, transferrin receptor type II, and ferroportin genes [7,8]. Secondary iron overload syndromes are due to various hematological disorders, chronic liver diseases, and porphyria cutanea tarda [9]. Iron overload represents a serious clinical condition since it may lead to the dysfunction of several organs, including the liver, heart, joints, skin, and endocrine glands, resulting in cirrhosis, cardiomyopathy, arthropathy, diabetes mellitus, hypopituitarism, hypothyroidism, and hypogonadism [10]. In particular, iron overload toxicity is mediated by cellular damage triggered by ferroptosis through reactive oxygen species (ROS) production through the Fenton reaction [11]. In this regard, previous reports showed that redox-active iron (i.e., non-transferrin-bound iron) is the main source of radicals and organic reactive species that are strongly related to oxidative damage and carcinogenesis [12]. Furthermore, several studies have also reported that iron overload can induce cell autophagy, the process by which eukaryotic cells are degraded and damaged macromolecules and organelles are salvaged [13,14]. Autophagy may represent a stress adaptation that suppresses apoptosis, whereas in other cellular situations, it constitutes an alternative cell-death pathway [13,14].

Several pharmacological strategies are currently available to manage iron overload even though some important issues need to be raised, such as high therapy costs, toxicity, and patient’s quality of life. In this regard, the use of commercially available antioxidants possessing iron chelation properties may represent good candidates addressing all the raised issues presented above. Furthermore, such an approach has the advantage of affecting the two main pathophysiological moments leading to iron toxicity (i.e., iron accumulation and ROS formation).

Alpha-lipoic acid (ALA), also known as thioctic acid and 1,2-dithiolane-3-pentanoic acid, is a naturally occurring substance existing in almost all types of prokaryotic and eukaryotic cells [15]. ALA exhibits both hydrophilic and hydrophobic properties, being widely distributed in plants and animals in cellular membranes and cytosol. ALA possess many biochemical functions, being able to act as an antioxidant and metal chelator, and to reduce the oxidized forms of other antioxidant agents, such as vitamin C, vitamin E, and glutathione (GSH) [15]. ALA has been also used for various chronic diseases, such as diabetes mellitus (DM) and its complications, hypertension, Alzheimer’s disease, Down syndrome, cognitive dysfunction, and some types of cancers [15,16].

The aim of the present study was to investigate the biochemical mechanisms underlying the protective effects of ALA in in vitro and in vivo models of iron overload.

## 2. Results

### 2.1. α-Lipoic Acid Chelating and Antioxidant Properties In Vitro

**Figure 1:**
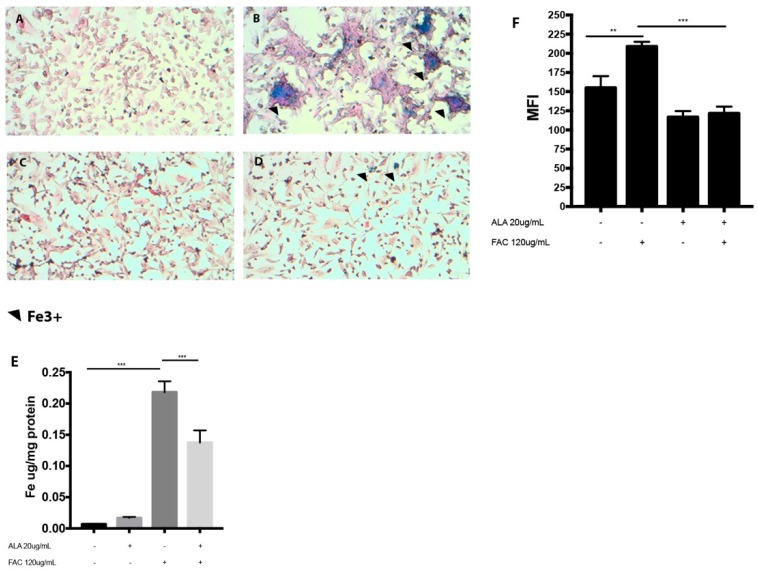
Perl’s staining in untreated HS-5 cell line (**A**) and following treatment with FAC (120 µg/mL) alone (for 24 h) (**B**) and with ALA (20 µg/mL) alone (**C**) or in combination with FAC (**D**); intracellular iron concentration assessment (**E**); reactive oxygen species reduction following co-treatment of FAC plus ALA (*** *p* < 0.0001) at 2 h in HS-5 cell line vs. FAC alone (**F**). Results are expressed as median fluorescence intensity (** *p* < 0.001 vs. untreated control and *** *p* < 0.0001 vs. FAC alone). All values are presented as mean ± SE of four experiments in duplicate.

**Figure 2:**
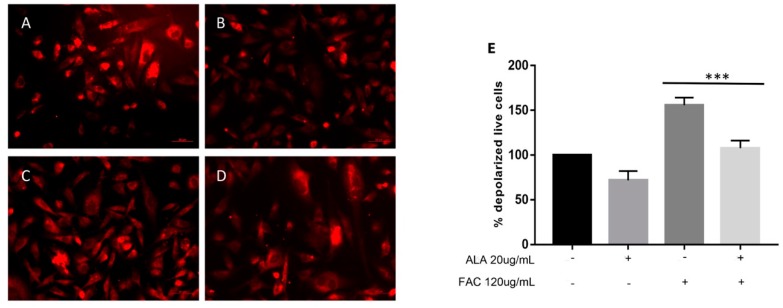
Immunofluorescences of TUFM localization in untreated HS-5 cell cultures (**A**) following FAC (120 µg/mL for 24 h) treatment alone (**B**) and with ALA (20 µg/mL) alone or in combination with FAC (**C**,**D**) and mitochondrial membrane depolarization evaluation (**E**). TUFM detection was performed by incubation with anti-goat monoclonal antibody followed by secondary antibody conjugated to Rhodamine (red). Counterstaining of cells was performed by using the nuclear dye, DAPI (blue); (Scale bars 10 µm). Mitochondrial membrane depolarization evaluation after FAC treatment alone and in combination with ALA performed by FACS analysis (*** *p* < 0.0001 vs. FAC alone treatment). All values are presented as mean ± SE of four experiments in duplicate.

Our results showed that FAC treatment (Figure 1B) results in a significant increase in intracellular iron content as measured following Perls staining when compared to untreated cells (Figure 1A). Furthermore, ALA treatment reduced iron storage when co-administrated with FAC (Figure 1D) compared to FAC alone (Figure 1B). These results were further confirmed by Inductively Coupled Plasma‒Optical Emission Spectrometer (ICP-OES) assay (Figure 1E). Consistent with these results, we also showed that iron overload resulted in a significant increase in ROS formation (Figure 1F) when compared to untreated cells and such an increase was cancelled by concomitant treatment with ALA. Our results also showed that FAC treatment resulted in a significant mitochondrial impairment as measured by a significant reduction of Tu translation elongation factor, mitochondrial (TUFM) expression (Figure 2A–D). These results were further confirmed by cytofluorimetric analysis demonstrating a significant loss of mitochondrial membrane potential (Figure 2E). Moreover, concomitant administration of ALA cancelled the detrimental effects of FAC when compared to FAC alone (Figure 2).

**Figure 3:**
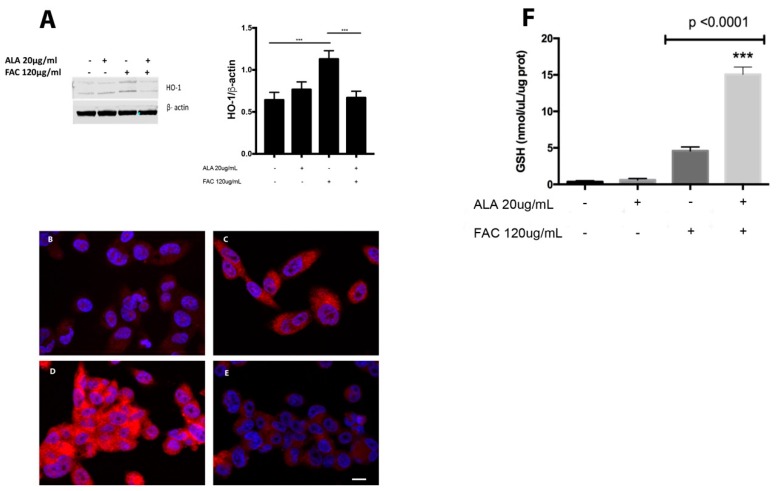
HO-1 protein levels in HS-5 cell cultures treated with FAC (120 µg/mL for 24 h) alone or in combination with ALA (20 µg/mL) were visualized by immunoblotting with specific antibodies (**A**). ß-actin shows an equal amount of protein loading in all lanes. Immunofluorescence showed HO-1 localization in untreated HS-5 cells (**B**) following treatment with FAC (for 24 h) alone (**D**) and with ALA alone or in combination with FAC (**C**,**E**). All values are presented as mean ± SE of four experiments in duplicate; (*** *p* < 0.0001) (Scale bars 10 µm).

Increased oxidative stress following FAC treatment led to a significant increase in heme oxygenase 1 (HO-1) protein expression when compared to untreated cells and such an increase was prevented by concomitant treatment with ALA (Figure 3A). These results were further confirmed by immunocytochemical analysis (Figure 3B–E). In addition, our results showed a significant increase in intracellular glutathione (GSH) content following FAC treatment when compared to untreated cells (Figure 3F). Interestingly, co-treatment with ALA and FAC resulted in a further significant increase of GSH content when compared to FAC alone or untreated cells.

### 2.2. In Vitro Effect of α-Lipoic Acid on Iron Overload-mediated Autophagy

**Figure 4:**
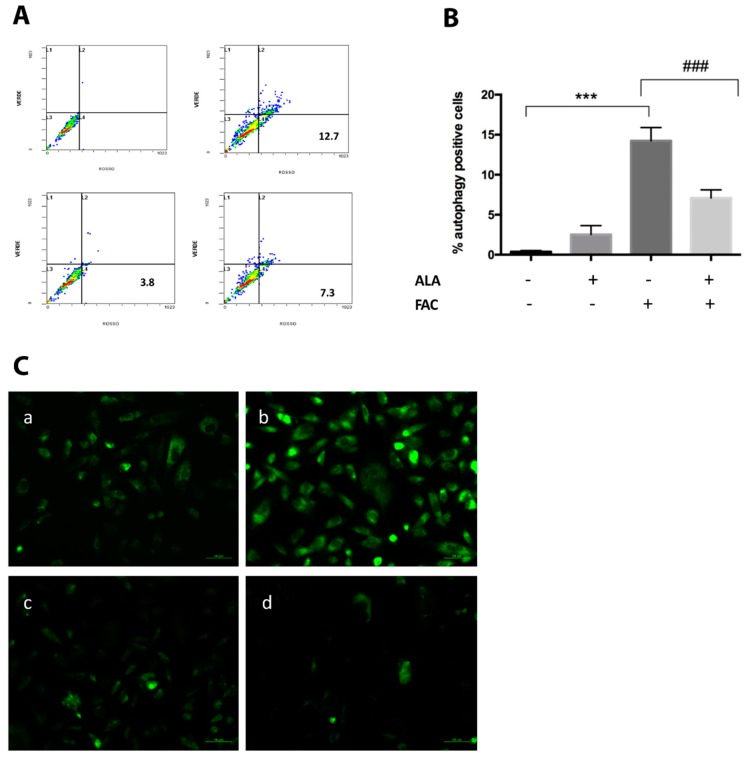
FACS analysis of autophagy induction in HS-5 cell cultures following FAC treatment (120 µg/mL) alone and in combination with ALA (20 µg/mL) (**A**,**B**). Results are presented as the percentage of positive cells to Acridine-orange staining (*** *p* < 0.0001 vs. untreated control; ^###^ *p* < 0.0001 vs. FAC alone treatment). The immunofluorescence image showed LC3-II localization in untreated HS-5 cells (**C**.**a**) following FAC treatment (**C**.**b**) and ALA alone (**C**.**c**) or in combination with FAC (**C**.**d**). All values are presented as mean ± SE of four experiments in duplicate.

Consistent with previous reports, our results showed that iron overload following FAC treatment results in a significant increase of autophagy as measured by the AVO test when compared to untreated cells (Figure 4A,B). Similar to oxidative stress results, co-treatment with FAC and ALA resulted in a significant reduction of autophagy when compared to FAC alone (Figure 4A,B). These results were further confirmed by immunocytochemical analysis showing that FAC treatment resulted in a significant increase of Microtubule-associated protein 1A/1B-light chain 3 (LC3-ΙΙ) when compared to controls (Figure 4A–D) and such an effect was prevented when FAC and ALA were co-administered.

### 2.3. In Vivo Effect of α-Lipoic Acid, Oxidative Stress, and Organ Injury

**Figure 5:**
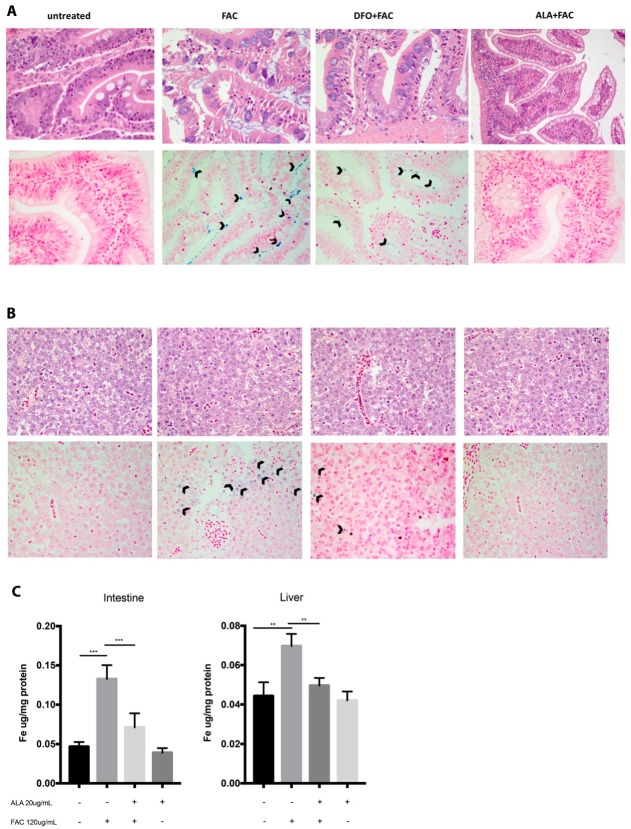
ALA protected against histopathological alterations and iron stores induced by iron overload in zebrafish intestine (**A**) and liver (**B**). Hematoxylin-Eosin staining showed histological damage induced by FAC (120 µg/mL for 48 h) treatment alone or in combination with ALA (20 µg/mL) or DFO (131 µg/mL). Perls staining was performed to detect iron stores following FAC treatment and in combination with ALA or DFO. Arrows indicate iron stores both in intestinal epithelium cells and hepatic cells. (Magnification 40×; scale bars indicate 50 µm). Iron level determination by ICP-OES (**C**). Results are expressed as the means ± SD of at least three independent experiments; ** *p* < 0.001, *** *p* < 0.0001.

Consistent with the in vitro results, we also showed that FAC treatment in a zebrafish model resulted in a significant increase in liver and intestine iron storage (Figure 5A,B) when compared to controls (Figure 5). In addition, our data showed that concomitant treatment with ALA prevented an increase in iron content in all examined tissues (Figure 5). Surprisingly, under our experimental conditions, no significant increase of iron storage was observed following FAC treatment (Figure 5). Iron storage reduction following ALA treatment was even more evident compared to DFO treatment (Figure 5A,B). Quantitative determination of iron (ICP-OES assay) showed that ALA reduced the iron storage in animals treated with ALA + FAC (Figure 5C).

**Figure 6:**
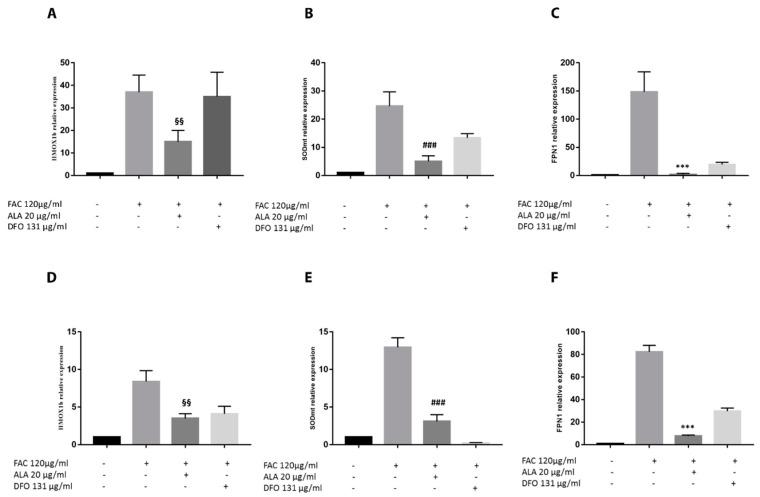
Effect of ALA on oxidative stress parameters of zebrafish liver and intestine. Gene expression analysis was performed following FAC treatment (120 µg/mL) alone and in combination with ALA (20 µg/mL) and DFO (131 µg/mL) for 48 h in zebrafish liver and intestine. HMOX1b, mtSOD (oxidative stress markers), and FPN1 levels were measured in the liver (**A**–**C**) and intestine (**D**–**F**) (HMOX1b: ^§§^ *p* < 0.001; mtSOD: ^##^ *p* < 0.001; FPN1: *** *p* < 0.0001 vs. FAC treatment). Calculated value of 2^ΔΔ*C*t^ in untreated controls was 1. Data are expressed as mean ± SD of at least three independent experiments.

These results were further confirmed by ferroportin 1 (FPN1) expression showing that iron overload following FAC treatment resulted in a significant upregulation of gene expression in the liver and intestine (Figure 6C,F) when compared to controls. ALA treatment resulted in a significant decrease of FPN1 gene expression when compared to treatment with FAC alone. In addition, no significant changes were observed in FPN1 expression compared to both concentrations of DFO in the liver (Figure 6F) whereas in the intestine, ALA resulted in a significant decrease of gene expression when compared to both DFO concentrations (Figure 6A–C). Concomitantly to the above-presented results, we also showed that FAC treatment resulted in a significant increase of oxidative stress as measured by heme oxygenase 1b (HMOX1b, *Danio rerio*) and mitochondrial superoxide dismutase (mtSOD) gene upregulation in the intestine and liver (Figure 6A,D). Our results again showed that ALA treatment resulted in a significant reduction of oxidative stress when compared to treatment with FAC alone. Finally, our results showed that oxidative stress reduction following ALA treatment was not significantly different when compared to DFO in the heart and liver, whereas it was significantly reduced in the intestine (Figure 6). As a result of increased iron deposition and oxidative stress following FAC treatment, morphological analysis of the intestine demonstrated the presence of clear features of organ injury (Figure 5). ALA treatment prevented organ injury in the intestine when compared to FAC treatment and histopathological recovery was significantly improved compared to DFO (Figure 5). No significant morphological abnormalities were observed in the liver following all pharmacological treatments (Figure 5).

## 3. Discussion

Iron overload may occur under various pathological conditions, including genetic forms, such as hereditary haemochromatosis, while others are acquired, such those related to repeated transfusions [17,18,19]. There are three chelating agents currently approved by the US Food and Drug Administration (FDA): Deferoxamine, deferiprone, and deferasirox. Iron chelators can reduce complications, such as cardiomyopathy, the major cause of death from iron overload. Furthermore, iron chelation therapy can attenuate the progression of liver fibrosis and glucose intolerance in transfusion dependent patients [20,21]. However, in more than 10% of patients, the use of such agents is associated with adverse effects, such as retinal and auditory neurotoxicity, neutropenia and agranulocytosis, diarrhea, headache, nausea, abdominal pain, increased serum creatinine, and increased liver enzymes, rash, fatigue, and arthralgia [22]. Therefore, the aim of the present study was to test the effect of ALA in in vitro and in vivo models of iron overload with particular regard to its antioxidant and iron chelating properties.

Previous studies suggested that the accumulation of mitochondrial iron contributes to the decay of mitochondria and decreases life-sustaining functions, such as adenosine triphosphate (ATP) production, intracellular Ca^2+^ buffering, regulation of cellular redox balance, and apoptosis [23,24]. Our in vitro results are consistent with these observations showing that FAC leads to intracellular iron accumulation, thus resulting in a significant impairment of mitochondrial membrane potential and organelle integrity, leading to ROS formation. Furthermore, our results showed that following administration of ALA, the levels of ROS gradually decreased toward basal conditions in mesenchymal stem cells, reducing intracellular iron content and restoring mitochondrial membrane potential and integrity. ALA is a dithiol compound normally bound to lysine residues of mitochondrial α-keto acid dehydrogenases; cytosolic and mitochondrial dehydrogenases rapidly reduce LA to dihydrolipoic acid (DHLA) [15]. Previous reports showed that ALA binds iron or any bivalent metal; hence, its property of iron chelation reduces the amount of free iron in the body, thereby alleviating oxidative stress, both enzymatically and by a free radical direct scavenging effect [15,25,26]. Interestingly, ALA is able to act inside the lysosomes, the conjugated action of cysteine and acid pH favors the rapid reduction of ALA in DHLA, possibly with the help of the lysosomal constituent, such as the lysosomal thiol reductase. Through its two vicinal thiolic groups, DHLA forms a Table 2:4 complex with Fe III (Fe_2_ [DHLA]_4_), and a less stable one with Fe II [27,28,29,30,31,32,33]. Several groups have reported that lysosomal degradation of ferritin is crucial for the utilization of ferritin iron stores in a number of different settings and plays a central role in iron extraction from ferritin. In this process, it was shown that DHLA removes iron from ferritin in vitro [29]. Our results were further confirmed by HO-1 expression and intracellular GSH content. The GSH system is the most important cellular defense mechanism as a ROS scavenger regulating the intracellular redox state [34] and its synthesis is promptly activated by various oxidative triggers. In this regard, Macias-Barragan J et al. [35] showed that cell exposure to Cd^2+^ resulted in a significant increase of GSH content as a result of the transcription activation of the enzymatic machinery for its biosynthesis. Furthermore, the authors showed that ALA also resulted in a significant increase of GSH content through the activation of the same pathway. Consistently with these observations, our results showed that iron overload following FAC treatment resulted in a significant increase in GSH content and that the concomitant treatment with ALA further increased such content when compared to FAC alone. Studies show ALA and DHLA may enhance cellular antioxidant defenses by a number of different mechanisms, including a direct antioxidant effect, and indirectly by augmenting the cellular GSH pool by increasing the expression of γ-glutamylcysteine ligase, the rate-controlling enzyme for GSH synthesis, Nrf2 activation [36]. In this regard, our resulted showed that HO-1, an NrF2 regulated protein involved in redox balance, is also upregulated following FAC treatment. Interestingly, co-treatment with FAC and ALA significantly reduced HO-1 expression when compared to FAC alone. These results may be dependent, at least in part, on the ability of ALA to increase intracellular GSH content, thus preventing NrF2 activation. Besides mitochondria, lysosomes are a major source of redox-active iron. Indeed, lysosomes are responsible for the autophagic degradation of iron-rich organelles, such as mitochondria and other metalloproteins. In this regard, our results also showed a significant increase in autophagy as measured by the increased number of autophagic granules following cytofluorimetric analysis and LC3 expression. Similar to our oxidative stress results, ALA treatment resulted in a significant decrease of autophagy. Finally, our in vitro results were further confirmed in an in vivo model of iron overload. These results were consistent with the in vitro results, showing that ALA results in a significant reduction of iron storage in the liver and intestine and in the expression of FPN1. It is noteworthy that ALA resulted in a significant reduction of FPN1 expression when compared to a clinically relevant concentration of DFO. Furthermore, ALA, because of its direct and indirect antioxidant properties, resulted in a significant reduction of HMOX1b and mtSOD expression in the heart and intestine when compared to DFO.

## 4. Materials and Methods

### 4.1. Cell Culture and Materials

Human mesenchymal stromal cell line, HS-5 (ATCC^®^ CRL-11882™), was maintained in Dulbecco’s Modified Eagle Medium-high glucose (Gibco, Thermo Fisher Scientific, Waltham, MA, USA), supplemented with 10% fetal bovine serum (FBS) (Gibco, Thermo Fisher Scientific, Waltham, MA USA), 10,000 U/mL penicillin, and 10 mg/mL streptomycin. Cells were grown to confluence and used from passages 4 through 7. Iron overload was obtained by ferric ammonium citrate (FAC, Alfa Aesar- TERMOFISHER) (120 µg/mL for 24 h). In a separate set of experiments, cells were pretreated (2 h) with 20 µg/mL of ALA to evaluate its antioxidant and chelating properties.

### 4.2. PerlsSstaining

Perls staining (Bio-Optica, Milan, Italy) was performed according to the manufacturer’s instructions. Briefly, tissue sections were passaged in distilled water and stained with Perls staining (Bio Optica, Milan, Italy) for 20 min. Sections were then rinsed in distilled water, dehydrated in ascending alcohols, cleared in xylene, and finally mounted for microscopic analysis.

### 4.3. Intracellular ROS Measurement

To determine the intracellular ROS generation (mainly superoxide), cells were stained with 5 mM dihydroethidium (DHE, Sigma-Aldrich, Milan, Italy) in PBS for 30 min at 37 °C. Fluorescence (excitation at 488 nm, emission at 620 nm) was determined by fluorescence-activated cell sorting (FACS, FC500, Beckman Coulter, Milan, Italy) [32]. N-acetyl cysteine 5 μM (NAC; Sigma-Aldrich) was used as a positive control of the ROS scavenger (data not shown).

### 4.4. Intracellular GSH Measurement

Intracellular content of reduced glutathione (GSH) was measured using a spectrophotometric assay based on the reaction of thiol groups with 2,2-dithio-bis-nitrobenzoic acid (DTNB) at *λ* = 412 nm (εM = 13,600 M^−1^·cm^−1^, where εM is a wavelength-dependent molar absorptivity coefficient). Measurements were performed in triplicate.

### 4.5. Immunofluorescence

Cells were grown directly on coverslips before immunofluorescence. After washing with phosphate-buffered saline (PBS), cells were fixed in 4% paraformaldehyde (Sigma-Aldrich, Milan, Italy) for 20 min at room temperature. After fixation, cells were washed three times in PBS for 5 min and blocked in Odyssey Blocking Buffer for 1 h at room temperature. Subsequently, the cells were incubated with primary antibody against HO-1 (anti-rabbit, Cat. No. BML-HC3001-0025, Enzo Life Sciences, Milan, Italy) at a dilution of 1:200 and LC3IIb (anti-rabbit, Cat. No. 398822, Santa Cruz Biotechnology, Milan, Italy) and TUFM (anti-goat, Cat. No 12990, Santa Cruz Biotechnology, Milan Italy) at a dilution of 1:200, overnight at 4 °C. Next day, cells were washed three times in PBS for 5 min and incubated with secondary antibodies: FITC (anti-rabbit, Cat. No. F0382, Sigma Aldrich, St. Louis, MI, USA) at a dilution of 1:200, and FITC (anti-rabbit, Cat. No. sc-2012, Santa Cruz Biotechnology, Santa Cruz, CA, USA) at a dilution of 1:200 and PE-cojugated (anti-goat, Cat. No. 3755) for 1 h at room temperature. All antibodies were diluted in Odyssey Blocking Buffer. The slides were mounted with medium containing DAPI (4′, 6-diamidino-2-phenylindole, Santa Cruz Biotechnology, Santa Cruz, CA, USA) to visualize nuclei. The fluorescent images were obtained using a Zeiss Axio Imager Z1 Microscope with Apotome 2 system (Zeiss, Milan, Italy).

### 4.6. Cytofluorimetric Analysis of Autophagy

Autophagic cells and formation of acidic vesicular organelles were quantified by FACS following acridine orange staining. Briefly, 20 µg/mL solution of acridine orange in appropriate buffer were added to100 µL of cell suspension and incubated at room temperature for 20 min. Finally, the appropriate isotopic control was also included and labeled cells were acquired using a Beckman Coulter FC-500 flow cytometer [33,34].

### 4.7. Mitochondrial Membrane Potential

Cells were seeded at 1 × 10^5^ cells/mL per plate in a 6-well plate and incubated for 24 h. Cellular mitochondrial membrane potential was assayed using the Muse MitoPotential Kit according to the user’s guide. A total of 1 × 10^5^ cells were collected by centrifugation (3000 rpm, 5 min) and washed with PBS. The supernatant was then removed and the cell pellets were stained with the Muse MitoPotential Kit (Merck Millipore, Guyancourt, France) for 25 min at 37 °C. The data was analyzed using the Muse™ Cell Analyzer Assay.

### 4.8. Western Blot Analysis

Briefly, for western blot analysis, 50 µg of protein was loaded onto a 12% polyacrylamide gel Bolt ^TM^ 8% Bis- Tris Plus Invitrogen (Thermo Fisher Scientific, CA, USA) followed by electrotransfer to nitrocellulose membrane iBlot^®^ Gel Transfer Stacks Nitrocellulose Regular (Thermo Fisher Scientific, Kiryat Shmona, Israel) using iBLOT Invitrogen transfer (Life Technologies, Israel). Subsequently, the membrane was blocked in Odyssey Blocking Buffer (Licor, Milan, Italy) for 1 h at room temperature. After blocking, the membrane was washed three times in PBS for 5 min and incubated with primary antibodies against HO-1 (1:1000) (anti-rabbit, Cat. No. BML-HC3001-0025, Enzo Life Sciences, Milan, Italy) and β-actin (1:1000) (anti-mouse, Cat. No. 69879, Santa Cruz Biotechnology, CA, USA) overnight at 4 °C. The following day, membranes were washed three times in PBS for 5 min and incubated with Infrared anti-mouse IRDye800CW (1:5000) and anti-rabbit IRDye700CW secondary antibodies (1:5000) in PBS/0.5% Tween-20 for 1 h at room temperature. All antibodies were diluted in Odyssey Blocking Buffer. The blots were visualized using an Odyssey Infrared Imaging Scanner (Licor, Milan, Italy) and protein levels were quantified by densitometric analysis of antibody responses. Data were normalized to the total protein levels of β-actin.

### 4.9. Animals

Adult wildtype AB zebrafish (*n* = 10, for each group) were used for this study. Fishes were tested to be free from *Pseudoloma neurophilia*, *Pseudocapillaria tomentosa*, *Mycobacterium spp*., and *Edwardsiella ictalurias* determined by twice-yearly sentinel monitoring. Fishes were housed at a density of 5 fishes per tank in mixed-sex groups in 2.5 L tanks on a recirculating system in 28 °C water in a room with a 14:10 h light:dark cycle. System water was carbon-filtered municipal tap water, filtered through a 20 µm pleated particulate filter, and exposed to 40 W UV light [35]. Standard feeding protocol was three meals daily of Tetra-Min (Tetra) in the CAPIR (University of Catania) facility. All zebrafish experiments were performed with the approval of the Animal Studies Committee of Ministero della Salute Italy (Approval code: 813/2017-PR, 23 October 2017).

Each experimental group (*n* = 10) was randomly assigned to a different treatment condition. FAC 120 µg/mL, ALA 20 µg/mL, and DFO (deferoxamine) 131 µg/mL (a commercial chelating drug used as a positive control), the chemical agents tested were introduced into a static 2 L tank filled with system water obtained from the main recirculating system. A behavioral control group of untreated fish housed under the same conditions as the experimental groups was tested in parallel. Experimental fish were monitored up to 48 h.

### 4.10. RNA Extraction and qPCR

RNA was extracted from dissected liver, intestine, gills, and heart by using TRIzol (Invitrogen). RNA was measured, and 1 µg of RNA with a 260/280 ratio>1.8 was used for reverse transcription by using a high-capacity cDNA kit (Applied Biosystem). PerfeCTa^®^ SYBR^®^ Green Supermix for iQ™ (Quanta Biosciences, Gaithersburg, MD) was used for real-time RT-PCR experiments in a FAST-HT 7900 Real Time PCR System (Applied Biosystem) under the following conditions: 95 °C for 3 min, 95 °C for 15 s/60 °C for 30 s (40 cycles). To ensure that only a single product was amplified, all real-time RT-PCR experiments were followed with a melt-curve analysis. For all experiments, GAPDH RNA was used as a ‘housekeeping’ gene for normalization. GAPDH RNA levels were not affected by any pro-oxidant treatments. The following sense and antisense primers (5′→3′) were used: Zebrafish HMOX1b sense: 5′-GCAGTGATCTGTCTGAACAG-3′, antisense 5′-GCTTGTACTGTGTTTGTGTG-3′; zebrafish mtSOD sense: 5′-ATGGCTTTAACATATCCGG-3′, antisense: 5′-TTCAGGGCTCAGGCTGG-3′; zebrafish FPN1: 5′-GGCCAGCACAGCTATGTC-3′ antisense: 5′-GCCAGAATGTTGGTCAAC-3′.

### 4.11. Morphological Analysis

Zebrafish intestinal mucosa tissues were collected and fixed in 10% buffered-formaldehyde; after an overnight wash, specimens were dehydrated in graded ethanol and paraffin-embedded, preserving their anatomical orientation. Three to four micrometer thick sections were obtained according to routine procedures, mounted on sialane-coated slides and air-dried. Slides were dewaxed in xylene, hydrated using graded ethanol, and stained for histological studies (Hematoxylin and Eosin and Perls staining).

### 4.12. Iron Level Determination

The samples (cellular pellet or homogenate) were digested overnight with 200 µL of Nitric Acid 65%, Suprapur^®^ for trace analysis (Carlo Erba). After digestion, ultra-pure water (Merck) was added to the samples up to a volume of 2 mL and iron (Fe) was quantified with an Inductively Coupled Plasma‒Optical Emission Spectrometer (ICP-OES Optima 8000, Perkin Elmer, USA). Standards for the instrument calibration were prepared on the basis of mono-element certified reference solution ICP Standard (Merck) in the same acid matrix of the samples as well as the calibration blank. The method detection limits (MDL) estimated with 10 blanks was 5.4 µg/L, calculated according to the following equation: MDL= One-tailed student’s *t*-test (*p* = 0.99%; df = *n* − 1) × Sr. A laboratory-fortified matrix (LFM) was determined as quality control and a recovery rate of 111% was obtained.

### 4.13. Statistical Analysis

Results are expressed as the means ± standard deviation (SD) of at least three independent experiments. Statistical analysis was carried out by one-way analysis of variance using the GraphPad Prism 4.0 software (GraphPad Software, San Diego, CA, USA). Differences were considered significant at *p* < 0.05.

## 5. Conclusions

In conclusion, ALA may represent a valuable tool to be used in iron overload conditions because of its pleiotropic mechanisms of action, impacting on various, important pathophysiological mechanisms involved in cellular dysfunction and organ injury.

## Abbreviations

ALA	α-lipoic acid
FAC	ferric ammonium citrate
ROS	Reactive Oxygen Species
GSH	Glutathione
HO-1	Heme Oxygenase-1
DFO	Deferoxamine
TUFM ICP-OES LC3-ΙΙ	Tu Translation Elongation Factor, Mitochondrial Inductively Coupled Plasma‒Optical Emission Spectrometer Microtubule-associated protein 1A/1B-light chain 3
